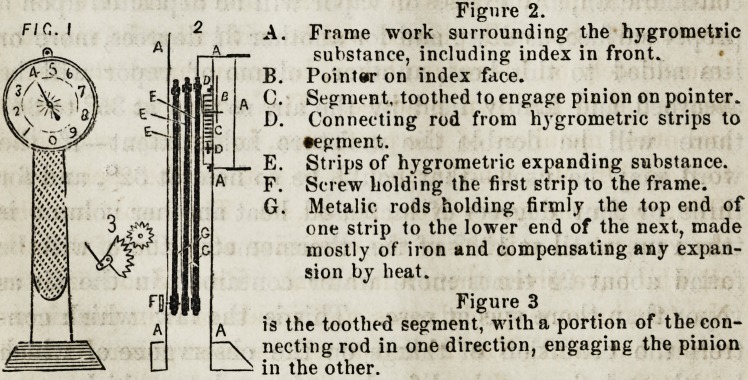# The Hygrometer

**Published:** 1870-03

**Authors:** 


### ARTICLE IV.

### *The Hygrometer,*

Or measurer of moisture, has hitherto attracted little or no popular attention, while as an instrument for hygienic objects, it has no equal in importance Doubtless the difficulty of constructing one, simple in its parts and accurate in registration, has prevented a popular and general knowledge of its usefulness, and lost to the people an unerring guide to the relations of heat, ventilation, and evaporation, and their combined effect upon health. A few words will recall to the recollection of such as are informed on the subject, the operation of the laws that control this instrument; which, though as simple as they are beautiful in themselves, seem to reach results by a complex process—but it is only seemingly complex—unless by dry calculation—as the hygrometer proves.

The principle of operation of the motor substance of a self registering instrument, is that it absorbs moisture from the surrounding air in which it is placed, until they—the substance and the air, become equally saturated and they so remain under all circumstances of change; the motor expanding with the absorption and contracting with the loss of vapor, incident to the amount of heat held from time to time. This expanding motion is communicated in some proper manner to the index, and the eye is instructed at a glance of the hygrometric condition. Now, when it is considered that the atmosphere holds vapor in quantity precisely proportioned to existing heat, but out of proportion in every *change* of temperature, it will be seen why the term "seemingly complex" has been used.

As a starting point assume a temperature of 32° Fah., thermometer; at this; as at all others within the range under consideration, any excess of vapor will be deposited upon a proper surface, as dew; and for another 3½ degrees, more or less added to this heat, another volume of vapor will be absorbed and firmly held by the air, so that at 35° to 36° there will be double the moisture held, latent—if the word may be used—that could be so held at 32°, and for three or four degrees of increased heat another volume is taken up, until at 100° of the thermometer there will be found about 25 times more water contained in the air as vapor than there was at zero. This is the law which controls the condition of things on the observance of which health and frequently life depends, and to which some unerring guide should unceasingly call attention.

In extremely dry atmosphere the tissues of the body, as indeed of all organic substances, are severely taxed in yielding to the despotic demand for moisture, but the principal danger to health is in the changes which occur during the reduction of the temperature of air highly charged with vapor. The law above hinted at operates inversely here, in that for three or four degrees of heat removed, what was called a volume of moisture at 32° is set free, and so for every additional reduction an additional volume is thrown in a chilled state upon all objects with which it comes in contact.

The importance of recognizing this truth for warm climates or high temperatures, will be conceded, when it is considered that four fifths of the vapor of water held at 100° is discharged before the mercury falls to 50°, rendering it to the animal body a danger of no mean significance and one which the senses do not always promptly recognize without artificial assistance.

The popular need for some simple and cheap instrument for ascertaining the quantity of moisture in the air, seems likely to be met by an ingenious apparatus before us, now being patented, invented by Mr. R. H. Atwell, of our city. It is useful for showing the condition of the air in sitting rooms, chambers or elsewhere, and will be fully understood from the following illustration and description.

Figure 2.

A. Frame work surrounding the hygrometric substance, including index in front.
B. Pointer on index face.
C. Segment, toothed to engage pinion on pointer.
D. Connecting rod from hygrometric strips to segment.
E. Strips of hygrometric expanding substance.
F. Screw holding the first strip to the frame.
G. Metalic rods holding firmly the top end of one strip to the lower end of the next, made mostly of iron and compensating any expansion by heat.

Figure 3

is the toothed segment, with a portion of the connecting rod in one direction, engaging the pinion in the other.

The mode of operation of this instrument is thus:

The strips E, which are generally of wood cut across the grain are inserted at their edges in shallow polished metalic grooves, and as moisture is absorbed by them, the movement commences at F. The first strip E, by its expansion imparts motion through rod G. to the next strip, which in turn sends forward the aggregate of its own and the movement behind it, and so on until at the rod D, connecting the last strip with the oscillating segment, (Fig. 3) a circular motion is communicated to the pointer B, of the index. This index instructs the eye by the engraved words "HEALTH," "DRY," "HUMID" or "DEW," as the case may be, and the numerals surrounding the face mark 100 degrees.

### SELECTED ARTICLES.

## Figures and Tables

**Figure f1:**